# Socio-economic determinants of attendance at diabetes self-management education program: using Andersen’s behavioral model

**DOI:** 10.1186/s12913-022-08749-x

**Published:** 2022-11-09

**Authors:** Javad Javan-Noughabi, Seyed Saeed Tabatabaee, Sajad Vahedi, Tahere Sharifi

**Affiliations:** 1 Social Determinants of Health Research Center, Mashhad University of Medical Sciences, Mashhad, Iran; 2 Department of Health Economics and Management Sciences, School of Health, Mashhad University of Medical Sciences, Mashhad, Iran; 3 Department of Health Care Management, School of Public Health, Ahvaz Jundishapour University of Medical Sciences, Ahvaz, Iran; 4 Social Determinants of Health Research Center, Institute for Future Studies in Health, Kerman University of Medical Sciences, Kerman, Iran

**Keywords:** Socioeconomic Factors, Diabetes Mellitus, Patient Acceptance of Health Care

## Abstract

**Background:**

Diabetes self-management education is an effective factor for improving outcomes and quality of life in patients with diabetes. However, little information is available on the factors associated with participation or non-participation in self-management education programs in people with diabetes. The aim of this study was to explore the factors affecting on the attendance of patients with diabetes in the diabetes self-management education program.

**Methods:**

A cross-sectional study was conducted in 2019 on 384 patients with diabetes referred to the main comprehensive health centers of Mashhad, Iran. All patients were linked with a diabetes self-management education program that lasted three months and involved 12 sessions. We explore the factors affecting on attending in diabetes self-management education program using Andersen’s behavioral model. Data for independent variables (predisposing, enabling, and need factors) were gathered at the beginning of the training program using registration forms. Dependent variable (attendance of patients with diabetes in the training program) was checked at the end of the program. Univariate and multivariate analysis were done with SPSS v.25.

**Results:**

The results of this study showed that women were less likely to participate in the self-management education program than men (OR=0.414; *P*<0.05). Also, age, travel time, health status and years with diabetes have negative significantly correlated with participation in the education program (*P*<0.05). The study showed that patients with diabetes aged≥65 were less participated in the training program than those ≤40 (OR=0.159; *P*<0.05). Also, patients who lived farther than 40 min away from training center were less likely to participate for this program than patients that live in an area<20 min away from training center (OR=0.196; *P*<0.05). Odds of attending in training program for patients with poor health status was less than patients with excellent health status (OR=0.282; *P*<0.05). Participation in training program were low in patients with more than 5-year diabetes duration compared to less than 1 year (OR=0.176; *P*<0.05).

**Conclusion:**

The implementation of the classes at the right time and online, Reduce the distance between people and the place of the class, providing facilities and providing infrastructure may be appropriate to involve women and the elderly.

## Background

Diabetes mellitus (DM) has been one of the most common chronic diseases for the last two decades [1]. According to estimates by the World Health Organization, if there is no managerial intervention, the number of people with the disease will increase from 346 million in 2018 to twice in 2030 [1]; And it is estimated that 9.2 million of them will be related to Iran [2]. The main reason for global attention to diabetes is its high prevalence and complications, because diabetes increases disability, reduces life expectancy, imposes heavy medical costs, and kills about 5 million people annually worldwide [3, 4].

In order to control diabetes and its complications, self-management is a useful and necessary strategy, because 95% of care is provided by the patient [5]. Control of the disease requires daily self-management, including the use of prescribed medications, regular self-monitoring, a healthy diet, and regular exercise [6]. Therefore, patients with diabetes and their families need to learn and practice new lifestyle skills [7].

In this regard, a simple concept called diabetes self-management education (DSME) is introduced, which means the promotion of self-determination and self-regulation [8]; And patients are encouraged to participate as much as possible in their treatment process by sharing information and cooperating in decision making [8]. The American Diabetes Association and the Academy of Nutrition and Dietetics also define DSME in a joint statement as a process that facilitates the knowledge, skills, and competencies necessary for diabetes self-management [9, 10]. As a result, DSME is recognized as an essential part of diabetes treatment [8]. To emphasize the need to teach self-management behaviors, it can be said that DSME is effective in improving the quality of life and reducing costs by preventing early and late complications of the disease and ensuring long life for the patient [8, 11].

However, several studies have confirmed the effectiveness of self-management education [12–14], but there are few studies about factors affecting on the attendance of patients with diabetes in the DSME programs [15, 16]. People that quit the DSME programs have worse health consequences and control of blood sugar than people who attend in these programs [11]. Currently, inconsistent and little information is available on the factors associated with attendance or non-attendance in DSME programs in people with diabetes. Boakye et al. in a similar study investigated the sociodemographic factors related to engagement in DSME in the United States [16]. However, their finding might be different in a developing country such as Iran. Therefore, the aim of this study was to investigate the factors affecting on the attendance of patients with diabetes in the DSME program based on the Andersen’s behavioral model of health service use in Mashhad, Iran.

### Conceptual framework

#### Andersen’s behavioral model of health service use

In this study, we applied the Andersen’s Behavioral Model of health service use. Anderson model of using health services that depends on individuals’ behavior is a theoretical model and used to study the background and personal features of those who have run into trouble utilizing health services. The objective of this model is to explain the reason of healthcare service use. This model is more far-reaching than others and considers health behavior affected by personal features divisible into three classes of predisposing, enabling and need factors. Predisposing factors are the status quo that may predispose one to use health services. Enabling ones are such factors as personal, familial and social features that can either facilitate or impede benefitting from services. Need factors refer to such factors as individuals’ general health and symptoms evaluated by health service providers [17–20].

## Methods

A cross-sectional study was carried out from 1 April to 1 July 2019 on patients with diabetes referred to the main comprehensive health centers of Mashhad. Mashhad is a metropolis in the northeast of Iran and the center of Khorasan-e Razavi province.

The sample size was 384, which has been determined with PASS (power analysis and sample size, PASS) software (Alpha=0.05, Beta=0.2).

$$N=\frac{{\left[{z}_{1-\frac{\alpha }{2}}\sqrt{\frac{\overline{P }\left(1-\overline{P }\right)}{R}}+{z}_{1-\beta }\sqrt{{P}_{0}\left(1-{P}_{0}\right)+\frac{{P}_{1}\left(1-{P}_{1}\right)\left(1-R\right)}{R}}\right]}^{2}}{{\left({P}_{0}-{P}_{1}\right)}^{2}\left(1-R\right)}$$

### Sample size formula

There are five main comprehensive health centers in Mashhad city (No. 1, 2, 3, and 5 and Samen Health Center). We selected 57 patients with diabetes from each center with convenience sampling. Diabetic patients diagnosed with pregnancy diabetes, glucose intolerance, unstable psychological state, or those who did not want to cooperate in the research project were excluded from the study. It should be noted that the sample size was evenly divided between the centers due to the relatively similar volume of population covered by the four selected centers. In this stage, we held a DSME program for all 384 patients with diabetes that lasted three months and involved 12 sessions. DSME program covered several main topics such as diet, physical activity, blood sugar monitoring, foot care and the management of the psychosocial issues. Non-attendance in DSME program defined as attending the classes fewer than three sessions [21]. We used Andersen’s behavioral model of health services use to predict factors that may facilitate or impede attending in the DSME program. In this model, factors are categorized into three groups. They are predisposing, enabling, and need factors that are used to predict health behaviors or health resource utilization. Predisposing factors are related to pre-existing sociodemographic characteristics; in this study, they include sex, age, education level, employment status and marital status. Factors that can facilitate or impede the use of services are named enabling factors. In this study, they include income/financial situation and geographical access. Need factors refer to the individual’s health status and need perceived by the individual. In this study, they include general health status and years with diabetes. Independent variables (predisposing, enabling, and need factors) were asked at the beginning of the training program using registration forms. At the end of the program, the number of meetings attended by each participant (dependent variable) was checked.

The data were analyzed by univariate and multivariate analysis. Univariate analysis was carried out to determine the frequency and percentage of variables. Finally, a logit regression model was used for multivariate analysis.

$${\mathrm{Y}}_{\mathrm{i}}=\alpha +{\beta }_{1}{X}_{i1}+{\beta }_{2}{X}_{i2}+{\beta }_{3}{X}_{i3}+\dots +{\beta }_{n}{X}_{in}+{u}_{i}$$

The dependent variable (Yi) is the probability of attending the DSME program. Yi = 1 for the patients who attended the three or more sessions of DSME program. Yi = 0 stands for the patients who attended the classes fewer than three sessions.

α shows the model intercept, n indicates the number of observations, ui refers to the random intervening component, and Bj = (j = 1, 2, 3, …, n) represent the model unknown parameters that must be estimated. In this equation, Xj stands for the model independent variables including sex, age, marital status, education level, employment status, household income, travel time, health status and years with diabetes.

## Results

The descriptive analysis is shown in Table 1. A total of 384 patients with DM were included in the present study. The majority of participants were female (52.3%), in the age group 41–64 years (39.3%), married (82.3%), with diploma and less education (59.9%), employed (74.5%), with middle household income (58.3%), less than 20 minutes away from the training center (40.4%), with very good health status (29.2%) and with 1-3 years history of DM (33.6%).

**Table 1:** Socio-demographic characteristics of patients

Variable		Health centers					Total (percent %)
		1	2	3	4	5	
Sex	Male	36	42	33	33	39	183 (47.7)
	Female	41	35	44	44	37	201 (52.3)
Age	≤40	24	25	13	25	15	102 (26.6)
	41-64	26	27	33	28	37	151 (39.3)
	≥65	27	25	31	24	24	131 (34.1)
Marital status	Single	21	5	0	12	30	68 (17.7)
	Married	56	72	77	65	46	316 (82.3)
Education level	Illiterate	12	4	1	5	1	23 (6)
	Diploma and less	37	48	46	46	53	230 (59.9)
	Academic education	28	25	30	26	22	131 (34.1)
Employment	Employed	49	52	67	58	60	286 (74.5)
	Unemployed	28	25	10	19	16	98 (25.5)
Household income	Low income	13	14	29	15	23	94 (24.5)
	Middle income	49	51	37	48	39	224 (58.3)
	High income	15	12	11	14	14	66 (17.2)
Travel time	<20 min	29	32	24	33	37	155 (40.4)
	20-40 min	18	23	32	24	23	120 (31.2)
	>40 min	30	22	21	20	16	109 (28.4)
Health status	Excellent	19	17	22	11	8	77 (20)
	Very good	13	22	23	21	33	112 (29.2)
	Good	19	20	17	22	16	94 (24.5)
	Fair	19	11	7	11	8	56 (14.6)
	Poor	7	7	8	12	11	45 (11.7)
Years with diabetes	<1y	22	15	14	7	14	72 (18.8)
	1-3y	22	32	20	21	34	129 (33.6)
	3-5y	7	13	28	31	17	96 (25)
	>5y	26	17	15	18	11	87 (22.6)
**Total**		**77**	**77**	**77**	**77**	**76**	**384 (100)**

R2, the coefficient of determination, is the relative power of the Probit and the Logit models. Model summary was shown in Table 2.

**Table 2:** Model Summary

-2 Log likelihood	Cox & Snell R Square	Nagelkerke R Square
337.705	0.386	0.518

Based on the results of the Table 3, insignificant Hosmer and Lemeshow goodness of fit test (*p*-value = 0.774) shows that the final binary logistic regression model was good fit.

**Table 3:** Hosmer and Lemeshow Test

Chi-square	Df	Sig.
4.846	8	0.774

Based on the results of the Table 4, sex was one of the strong predictor of the participation in the DSME program. The study showed that women were less likely to participate in the DSME program than men (OR=0.414; *P*<0.05). The results of the logit model analysis indicate that the probability of participating in DSME program decreases with age. The study showed that patients aged≥65 were less likely to participate in DSME program than those ≤40 (OR=0.159; *P*<0.05). Also, patients that live in an area >40 min away from training center were less likely to participate for this program than patients that live in an area<20 min away from training center (OR=0.196; *P*<0.05). The study also revealed that odds of attending in training program for patients with poor health status was less than patients with excellent health status (OR=0.282; *P*<0.05). Participation in training program were low in patients with more than 5-year diabetes duration compared to less than 1 year (OR=0.176; *P*<0.05).

**Table 4:** Multivariate logistic regression model

Variable	Category	OR	95% CI		*P*-value
			Lower	Upper	
Sex	Male				
	Female	0.402	0.231	0.700	0.001
Age	≤40				
	41-64	0.207	0.099	0.433	0.000
	≥65	0.168	0.073	0.389	0.000
Marital status	Single				
	Married	0.569	0.271	1.195	0.136
Education level	Illiterate				
	Diploma and less	0.872	0.256	2.969	0.826
	Academic education	0.976	0.261	3.651	0.971
Employment	Employed				
	Unemployed	1.669	0.796	3.498	0.175
Household income	Low income				
	Middle income	2.240	1.117	4.493	0.023
	High income	1.506	0.611	3.713	0.374
Travel time	<20 min				
	20-40 min	0.276	0.147	0.520	0.000
	>40 min	0.198	0.099	0.398	0.000
Health status	Excellent				
	Very good	0.373	0.175	0.797	0.011
	Good	0.270	0.118	0.616	0.002
	Fair	0.228	0.075	0.691	0.009
	Poor	0.255	0.075	0.865	0.028
Years with diabetes	<1y				
	1-3y	1.191	0.558	2.545	0.651
	3-5y	0.488	0.220	1.083	0.078
	>5y	0.142	0.047	0.429	0.001

## Discussion

This study conducted aimed to investigate the determinant factors related to participation in the DSME program for patients with diabetes. In this study, we found that the participation rate was 43%. Previous studies conducted in developed countries showed that the participation rate varies between about 40% and 55% [16, 21–23]. These studies have identified a number of factors that may influence individuals’ decisions to attend training programs. These factors may vary between countries, so it is very important to identify and remove these obstacles. In this study, we assessed the effects of predisposing, enabling and need factors on the participating in the DSME program for patients with diabetes based on the Andersen’s Behavioral Model of health service use.

### Predisposing factors

Our study showed that the probability of participating in the DSME program decreases with age. Our results are consistent with previous studies, where participation in education programs was found to be lower amongst older adults [24, 25]. Older patients experienced a wide range of physical, mental and social health problems and they need extensive supports [26]. Fan et al. in their study reported a significant relationship between age and the type of DSME interventions. Older patients want comprehensive self-management courses that include a combination of educational, behavioral and psychological interventions [27]. Rhee et al. revealed that increasing age was an obstacle to participation in diabetes education [28]. Gucciardi et al. in their study in Canada concluded that patients with diabetes aged over 65 years were more likely to abandon education program than other age groups. It is consistent with our findings [21]. However, several studies showed different findings. Boakye et al. in their study in United States showed that patients with diabetes aged 65 were more likely than respondents aged 18-54 to engage in self-management education program [16]. Also, Cauch-Dudek et al. in a study in Canada reported that younger patients with diabetes were more likely to attend a diabetes self-management education program than older patients [24]. One of the reasons for the differences in the findings could be that the elderly have more free time and less busy in developed countries. Also, one of the reasons for older people to leave the training course in the present study could be because the classes did not meet their needs. Due to old age and complications of the disease, the elderly need more comprehensive and extensive information that should be considered in designing training courses for the elderly.

The findings of the current study showed that participating the educational services of self-management schemes was meaningfully lower among women than men. The findings of various studies also confirm the relationship between self-care behavior in patients with diabetes and gender. Boakye et al. in their study concluded that men were less likely than women to engage in diabetes self-management education [16]. It is inconsistent with our results that could be due to cultural and familial backgrounds and the distribution of responsibilities among family members. Woman tend to attend courses with active participation that involved face-to-face interactions, discussion and sharing information with diabetes educators [29, 30]. Therefore, for women’s participation in self-care programs, special attention should be paid to these points in designing the course. Thus, it is suggested that while performing such educational schemes, women’s conditions be noted. Furthermore, using supplementary educational programs, distance education, holding classes at proper times and short hours can help alleviate these obstacles.

It has been proven that virtual education for self-care programs can improve the health behaviors of consumers and decrease the workload of providers [15, 31]. Online training using common platforms in Iran such as WhatsApp can be effective in increasing the participation of women in the program. However, there may be some barriers such as loss of privacy, addiction, language and digital illiteracy to using virtual space. Therefore, the use of virtual space and online platforms requires the creation of necessary infrastructure, increasing digital literacy and promoting the culture of use.

The present study did not confirm a statistically significant relationship between education level and participation in the DSME program. However, previous studies showed different results. It is might be due to different population study. These studies revealed a statistically significant positive relationship between the education level and completing the training courses for diabetes [24, 25]. Kim et al. in their study in Korea showed that individuals with elementary school education or less were 3 times more likely not to attend training program relative to those with higher education level [32]. Rhee et al. confirmed that non-participation rate for patients with an elementary school education or less were 5 times higher than others [28]. Patients with higher levels of education preferred to acquire standard information about self-management through discussion with diabetes educators. Also, people with higher education level had more self-care behaviors such as blood sugar control [27, 32–34].

The results of the logit regression showed no statistically significant difference in attending DSME programs between Unemployed and employed patients. It is consistent with study conducted by Kim et al [32]. However, several other studies showed that working full and part-time were a main factor for inability to attend in diabetes education program. They concluded that conflict between work-time and time for training classes is a substantial factor for non-participation in these programs [21, 28, 35]. The difference in findings may be due to the heavy role and responsibility of housewives in Iran. Although housewives are unemployed, they were reluctant to attend classes due to housework and childcare. Therefore, the use of public media such as television and radio and the design of self-care training software for mobile phones will greatly enable people with different jobs to benefit from training.

### Enabling factors

In this study, we did not find a statistically significant relationship between household income status and participation in self-care classes. Previous studies showed that low-income patients were less likely than higher-income respondents to engage in a diabetes education program. Patients with lower socio-economic status may have lower levels of health literacy and more financial barriers to joining educational program than those with higher socio-economic status [16, 24, 25, 36]. The difference in findings may be due to different sample.

In this study, time interval has also been suggested as an effective factor in the participation of patients with diabetes in the DSME programs. The results of this study show that by increasing the distance from the training center, the participation of the patients in self-care programs will be significantly reduced. Previous studies showed that long distance from the education center was an obstacle for participation in training programs [21, 37].

### Need factors

The results of our study indicate that leaving the training program was more common with reducing health status. These results are supported with previous research studies. Patients with poor health status experience more physical and psychosocial problems than those with excellent health status. Such problems for patients with diabetes affect their mood and behavior and can lead to reduce their participation in the DSME program. It is claimed that worsening glucose control leads to worsening learning activities [38]. However, Gucciardi et al. in a multivariate logistic regression found that fewer diabetes symptoms was determinant factor to non-participation in education programs [21]. The difference in findings may be due to different sample.

In our study, participation in training program were less likely in patients with more than 5-year diabetes duration compared to those with less than 1 year. However, Kim et al. in their study on Korean patients with diabetes showed different results. They found a positive relationship between diabetes duration and participation rate in education program [32]. Another study in the Netherlands reported that short diabetes duration associated with low participation of patients with diabetes in self-management programs [39]. They argue that worse health conditions increase their concerns and can lead to participation in education program. One of the reasons for the difference in findings may be that people in developing countries do not pay much attention to education. In other words, there is a therapeutic focus. Also, Training classes may not be of the required quality to address the concerns of patients with a poor health condition.

## Conclusion

The results of this study showed that attending at the DSME program is influenced by predisposing factors (sex, age), enabling factors (geographical access) and need factors (general health status and years with diabetes). The implementation of the classes at the right time and online, reduce the distance between people and the place of the class, providing facilities and providing infrastructure may be appropriate to involve women and the elderly.

## Data Availability

The datasets generated and/or analysed during the current study are not publicly available due to protecting individual patient privacy and data protection regulations, but are available from the corresponding author on reasonable request.
